# Gender differences of aortic wave reflection and influence of menopause on central blood pressure in patients with arterial hypertension

**DOI:** 10.1186/s12872-018-0855-8

**Published:** 2018-06-19

**Authors:** Valeria Aparecida Costa-Hong, Henrique Cotchi Simbo Muela, Thiago Andrade Macedo, Allan Robson Kluser Sales, Luiz Aparecido Bortolotto

**Affiliations:** 1 Heart Institute (Incor), Hypertension Unit, University of São Paulo Medical School, 2nd floor, Room 8, Av. Dr. Enéas de Carvalho Aguiar, São Paulo, 44 – Cerqueira César 05403-900 Brazil; 2 Department of Physiology, Faculty of Medicine, Agostinho Neto University, Luanda, Angola; 3 Heart Institute (Incor), Cardiovascular Rehabilitation and Exercise Physiology Unit, University of São Paulo Medical School, São Paulo, Brazil

**Keywords:** Arterial hypertension, Menopause, Pulse wave reflection, Central aortic blood pressure, Augmentation index, Arterial stiffness

## Abstract

**Background:**

Evidences suggest that central hemodynamics indexes are independent predictors of future cardiovascular events and all-cause mortality. Multiple factors have been pointed to have potential influence on central aortic function: height, heart rate, left ventricular ejection duration and blood pressure level. Data related to the influence of gender and postmenopausal status on aortic waveform reflection is scarce. We aim to evaluate the impact of gender and menopause on central blood pressure of hypertensive patients.

**Methods:**

In a cross sectional study 122 hypertensive patients (52 men and 70 women) were studied. Hypertension was defined as blood pressure (BP) levels ≥140/90 mmHg or use of antihypertensive drugs. Central arterial pressure, augmentation index (AIx) and augmentation index normalized to 75 bpm (AIx75) were obtained using applanation tonometry. Menopause and postmenopause history were accessed by a direct series of questions. Postmenopause was defined as at least one year since last menstruation. Patients were paired by age, gender and menopausal status, and the data were compared considering gender and menopausal status.

**Results:**

Height and weight were significantly lower in women than in men at the same age. Conversely, AIx (32.7 ± 9.8% vs. 20.1 ± 11.7%, *p* < 0.01), AIx75 (29.6 ± 6.7% vs. 18.3 ± 9.4%, *p* < 0.01) and central systolic blood pressure (136 ± 30 vs. 125 ± 23 mmHg, *p* = 0.03) were higher in women than men. The menopausal women (mean age of menopause = 48 years) had the worst indexes of aortic wave reflection, compared to men at the same age and younger women.

**Conclusion:**

Hypertensive women had both higher reflected aortic pressure waveform and central blood pressure indexes than hypertensive men, and these findings were worsened by the menopausal status.

## Background

Evidences suggest that central hemodynamics indexes are independent predictors of future cardiovascular events and all-cause mortality. Central pressures do not exactly correspond to brachial pressures because of pressure pulse amplification from the aorta to the peripheral arteries [1]. Compared to peripheral pressure, central blood pressure has been shown to be a better predictor of cardiovascular disease and holds a prognostic value for both cardiovascular fatal and non fatal events. The augmentation index (AIx) is a variable obtained by analysis of pulse waves acquired by applanation tonometry, and basically reproduces an increase of the pressure waveform resulting from backward reflected wave from periphery to the aorta. It is shown that AIx can predict clinical events independently of peripheral pressures [1]_._ Multiple factors have potential influence on large arteries functional properties: height [2, 3], heart rate [3], left ventricular ejection duration [4] and blood pressure level [3, 5]. However, information about the influence of gender and postmenopausal period on these properties is scarce. We aimed to evaluate the influence of gender and menopause status on central aortic pressure, aortic wave reflection and carotid artery properties in hypertensive patients.

## Methods

### Subjects

We enrolled 122 (52 men and 70 women) patients from approximately 468 individuals screened for this cross-sectional study. Patients from the Hypertension Unit of the Heart Institute of University of São Paulo (Brazil) were consecutively recruited from June 2013 to June 2015.

The local ethics committee approved the protocol, and all participants agreed and signed the Informed Consent Form. The vascular evaluation was performed in the clinical research laboratory at the Heart Institute, room temperature controlled between 22 °C and 24 °C.

Menopause and postmenopause history were assessed by a direct series of questions (1. Do you still have menstrual period? 2. If not, when did it stop? 3. How old were you when your menstruation period stopped?). Postmenopause was defined as at least one year since last menstruation [6]. Because the minimum age reported for the menstruation cessation was 48 years old, this was stablished as the cutoff value to classify the women according to the menopause status. Based on this cutoff value for women and in order to compare them to men of the same age group, the sample was divided into 4 groups: group 1 (young men, ≤48y), group 2 (young women, ≤48y), group 3 (older men, >48y) and group 4 (older women, >48y). Patients with the following conditions were not included: age less than 18 years old, cerebrovascular disease (previous stroke or transient ischemic attack), diabetes mellitus, smoking, arrhythmias, heart failure with left ventricular dysfunction and known neurodegenerative or psychiatric disease.

### Measurements and procedures

#### Blood pressure measurements

Brachial systolic and diastolic blood pressure was measured using the Omron automatic device (HEM-705 CP model), on the arm with the highest BP, after 5 min of resting. The mean of 3 measurements with a 1-min interval between each one was obtained for both systolic and diastolic blood pressure (SBP and DBP) according the recommendations of the VI Brazilian Guidelines on Hypertension. Hypertension was defined by SBP ≥ 140 mmHg and/or DBP ≥ 90 or use of antihypertensive drugs [7].

#### Pulse wave analysis

The assessment of arterial pulse wave components was performed by a non-invasive applanation tonometry of radial artery a SphygmoCor device (AtCor Medical, Sydney, Australia). Radial artery pressure waveforms were recorded in the left wrist using a pencil-type high-fidelity micromanometer (Millar Instruments, Houston, Texas). The reliability and reproducibility of this technique has been previously reported [8–10]. The radial artery pressure curve was calibrated using brachial blood pressure. From the pulse wave analysis of the aortic pressure waveform the following variables were obtained: aortic pressures, aortic augmentation index (AIx), AIx adjusted for a heart rate of 75 beats per minute (AIx75) [11]. Aortic pressure waveform and augmentation index (AIx) were calculated by using the transfer function of SphygmoCor device. Aortic AIx was calculated as follows: AIx = ∆P/PP, ∆P=P2-P1 (P2: peak systolic pressure, P1: inflection point that indicates the beginning upstroke of the reflected pressure wave). Only high-quality recordings, defined as an in-device quality index of > 80% were accepted for analysis.

#### Carotid artery properties

Carotid measurements were obtained in the right common carotid artery by a 7.5 MHz linear array probe 1 cm below the bifurcation at the site of distal wall. Carotid diameter and distension were recorded using a high-resolution echo-tracking system (Wall Track, Pie Medical System, Maastricht, Netherlands), which is based on a radio-frequency signal analysis previously validated and used in different clinical studies [12, 13]. Briefly, a radio-frequency signal of 4–8 cardiac cycles was recorded, digitized, and temporarily stored. The signals corresponding to the proximal and distal walls were defined and the posterior wall thickness and the internal diameter were measured by positioning markers in the respective posterior and anterior wall signal. The system computed the successive values of internal end-diastolic diameter and stroke change in diameter and digitized the displacement waveform [12]. Carotid distension was automatically calculated as the systolic-diastolic variation.

### Statistical analyses

Data were analyzed with SPSS software (SPSS for Windows 20.0, SPSS, Inc., Chicago, IL). Data distribution was determined using the Kolmogorov–Smirnov test. Continuous variables are presented as mean and standard deviation and were analyzed by the independent-samples t test and Mann–Whitney U test, when suitable. Categorical data are presented as percentages and analyzed by Chi-square test. An ANOVA test was used with Bonferroni post hoc comparisons to analyze differences between gender and age groups. The AIx, AIx 75 and brachial SBP were adjusted for height and weight and analyzed by an ANCOVA test. Pearson’s coefficient was used for bivariate correlations. Statistical significance was set at 5%.

## Results

**Fig. 1:**
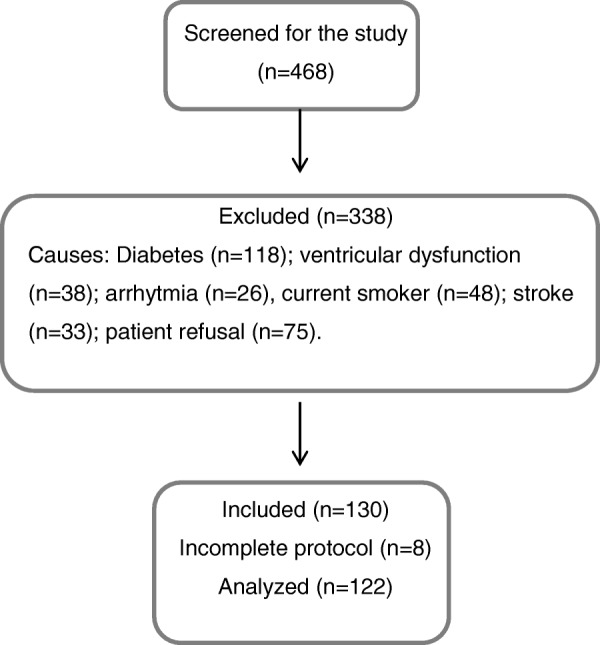
Flow chart of the study population

In a cross-sectional study we evaluated 122 hypertensive patients. The average duration of hypertension was 9.99 ± 7.9 years and 47.1% had BP levels under control (< 140/90 mmHg). The Fig. 1 shows the flow chart of the study and the main exclusion reasons. Clinical characteristics according the gender are described in Table 1. Height and weight were significantly lower in women than in men at the same age. Conversely, some variables values were significantly higher in women than in men, such as: AIx (32.7 ± 9.8% vs. 20.1 ± 11.7%, *p* < 0.01), AIx75 (29.6 ± 6.7% vs. 18.3 ± 9.4%, *p* < 0.01) and central systolic blood pressure (136 ± 30 vs. 125 ± 23 mmHg, *p* = 0.03), respectively. There was a negative correlation between radial AIx (*r* = − 0.48, *p* < 0.01) and AIx75 (*r* = − 0.55, *p* < 0.01) with the height of patients.

**Fig. 2:**
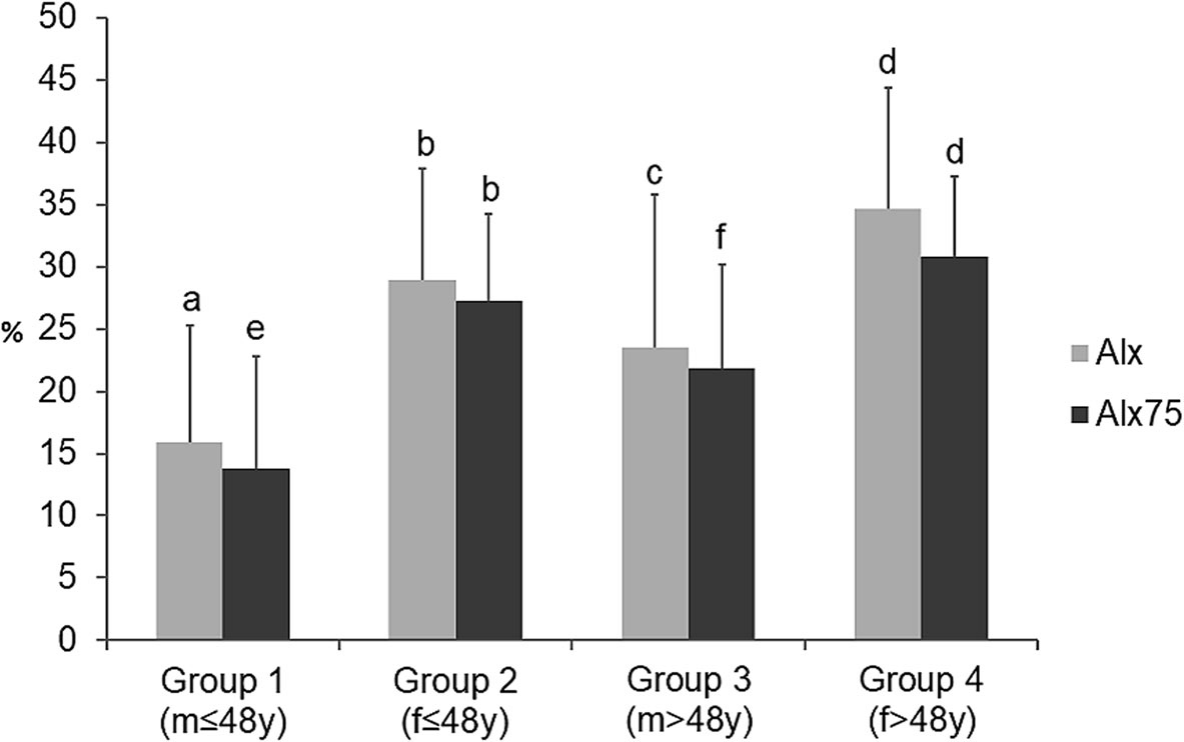
Values of aortic augmentation index (AIx) and AIx adjusted for a heart rate of 75 (AIx75) according to respective group. (m:male; f:female). **a)** group 1 vs. group 2 and group 4 (*p* < 0.01); **b**) group 2 vs. group 1 and group 4 (*p* < 0.01); **c**) group 3 vs. group 4 (*p* < 0.01); **d**) group 4 vs. all (*p* < 0.01); **e**) group 1 vs. all (*p* < 0.01);**f**) group 3 vs. group 1 and group 4 (*p* < 0.01)

**Table 1:** Clinical characteristics distributed by gender

Variables	men (*n* = 52)	women (*n* = 70)	*p*
Age (years)	49.7 ± 10.5	52.6 ± 11.0	0.15
SBP (mmHg)	137 ± 20	145 ± 26	0.06
DBP (mmHg)	86 ± 12	87 ± 15	0.60
HR	71.4 ± 13	68.2 ± 11.9	0.18
Weight (kg)	85.3 ± 11.4	76.2 ± 14.0	< 0.01*
Height (m)	1.72 ± 0.08	1.59 ± 0.06	< 0.01*
Hypertension time (years)	9.4 ± 7.1	10.8 ± 8.6	0.35
AIx (%)	20.1 ± 11.7	32.7 ± 9.8	< 0.01*
AIx75 (%)	18.3 ± 9.4	29.6 ± 6.7	< 0.01*
C_SP (mmHg)	125 ± 23	136 ± 30	0.03*
C_DP (mmHg)	88 ± 15	88 ± 16	0.90

*SPB* systolic blood pressure, *DBP* diastolic blood pressure, *HR* heart rate, *AIx* aortic augmentation index, *AIx*75 aortic augmentation index adjusted for heart rate of 75, *C_SP* central systolic blood pressure, *C_DP* central diastolic blood pressure. (*) Differences in AIx (22.2 ± 12.7 vs 30.9 ± 12.2) and AIx75 (20.5 ± 9.41 vs 27.7 ± 8.12) persist even after adjustments for: height 1.64 m, weight 80.1Kg, SBP 142 mmHg *p* < 0.01

Differences in AIx (22.2 ± 12.7 vs. 30.9 ± 12.2, *p* < 0.01) and AIx75 (20.5 ± 9.41 vs. 27.7 ± 8.12, *p* < 0.01) between women and men were maintained even after adjustment for height, weight and SBP. There were no differences among the groups according to hypertension duration and blood pressure level.

Menopause was registered in 70% of women. Young men (group 1) had lower AIx than both young (group 2) and older women (group 4) (16.5 ± 9.93 vs. 27.3 ± 8.74, 34.9 ± 9.77, *p* < 0.01) and the lowest AIx75 (14.5 ± 6.98) than all other groups respectively: young women (25.4 ± 7.87), older men (21.8 ± 8.53) and older women (30.9 ± 6.57). Conversely, young women (group 2) had both AIx and AIx75 comparable to older men group (AIx: 27.3 ± 8.74 vs. 23.2 ± 12.4, *p* = 0.9 and AIx75: 25.4 ± 7.87 vs. 21.8 ± 7.87, *p* = 0.7, respectively). When all groups were compared, the menopausal women (group 4) had the worst indexes of aortic wave reflection (AIx: 34.9 ± 9.77 (group 4) vs. 16.5 ± 9.93 (group 1), 27.3 ± 8.74 (group 2), 23.2 ± 12.4 (group 3), *p* = < 0.01 and AIx75: 30.9 ± 6.57 (group 4) vs. 14.5 ± 6.98 (group 1), 25.4 ± 7.87 (group 2), 21.8 ± 8.53 (group 3), *p* = < 0.01) (Fig. 2). There were no differences among groups according to values of brachial blood pressure (SBP *p* = 0.3; DBP *p* = 0.6).

**Fig. 3:**
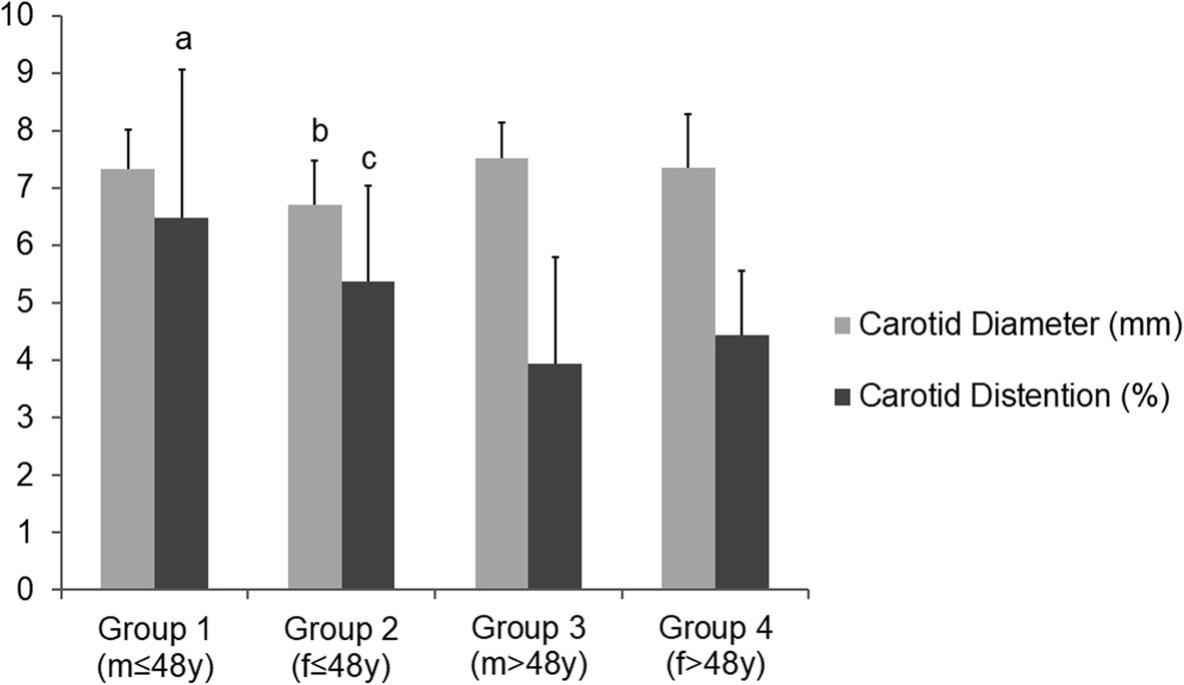
Values of carotid diameter and distention according to respective group. (m:male; f:female). **a**) group 1 vs. group 3 and group 4 (*p* < 0.01);**b**) group 2 vs. all (*p* < 0.01);**c**) group 2 vs. group 3 and group 4

Young women (group 2) had the lowest carotid diameter (6.68 ± 0.74 vs. 7.37 ± 0.69 (group 1), 7.49 ± 0.63 (group 3), 7.38 ± 0.92 (group 4), *p* < 0.01) (Fig. 3). Younger men and women groups (group 1: 6.12 ± 2.62 and group 2: 5.71 ± 1.91) had higher carotid distention than their older pairs (group 3: 4.01 ± 1.85 and group 4: 4.41 ± 1.13, p < 0.01). (Fig. 3).

Concerning the use of antihypertensive drugs that could possibly interfere in central aortic pressures, including beta-blockers, no significant differences were observed among the four groups.

## Discussion

The main finding of our study is that indexes related to an increase of wave reflection, radial AIx and AIx75, were higher in women than in men with hypertension, even after adjustments for the main recognized variables related to reflected waves as body height, weight and brachial SBP. Furthermore, postmenopausal women showed the highest indexes, compared to men at the same age or younger women.

Arterial wave reflection originates from every branching of arterial vessels. The aortic length matches with body height, thus arterial wave reflection occurs later in taller individuals [14, 15]. The height is one of the possible variables that could explain the elevated values of augmentation index in women compared to men in our population, because on average women are shorter than men. Women were on average 13 cm shorter than men in our sample, but the differences between men and women in AIx and AIx75 persisted even after adjustment for height. Moreover, there was a negative correlation between AIx and height, and there were no differences on blood pressure or on age that could explain the high values of Alx and AIx75 in women compared to men.

Gatzka et al. [16] studied elderly untreated hypertensives including 104 pairs of men and women with identical body height. The authors demonstrated that arterial wave reflection occurred earlier during systole in women than in men with the same height. The study also showed that women had more reduced arterial diameter and an increased arterial stiffness when compared to height-matched men, which cause the reflected wave to travel faster and arrive in central arteries earlier with greater amplitude and duration. In our study, although women had lower height than men at the same age, AIx, AIx75 and central systolic BP were higher, related probably to a faster reflected wave resulting in an increased augmentation index.

It has been demonstrated an increase in left ventricle pulsatile afterload [17] and changes in central (carotid) arterial pressure waveform [18] with aging, due to aorta stiffening. In our study, young women had smaller carotid diameter than other groups, thus resulting in an earlier return of the reflected arterial wave and greater augmentation index than men at the same age. Two studies had shown that women had smaller diameter and stiffer arteries which increased pulse wave velocity, resulting in an earlier return of the reflected arterial wave, an increased pulse pressure, and greater augmentation index [16, 19].

Evidence have shown that menopause has a negative influence on atherosclerotic risk factors [20] and amplifies the age-dependent increase in arterial stiffness [21]. Menopause was present in a high percentage (70%) of women in our sample. Zaydun G et al. [22] have suggested that, in the early postmenopausal phase, estrogen deficiency may increase the age-related arterial stiffness. Although studies have demonstrated an association between age and arterial stiffness, many studies did not investigate the prevalence of the menopause and also did not evaluate arterial stiffness in a narrow range of age (such as postmenopausal period) [23, 24]. Furthermore, there were no difference on BP and age and even after adjusting for height and weight, the menopausal women group still showed the highest values of AIx and AIx75, suggesting a possible influence of menopause on arterial stiffness.

### Limitations

The cross-sectional nature of this trial does not allow us to evaluate whether menopause has a direct role on aortic wave reflection and central blood pressure. Also, the patients were not submitted to echocardiogram, so correlations between left ventricular function and reflection indexes were not possible to evaluate. We did not have information about hormone replacement in postmenopausal women. Our study was carried out in a selected group of patients with hypertension referred to a tertiary hospital, limiting the application of our findings to other populations.

## Conclusion

Hypertensive women had an increased reflected aortic pressure waveform and a higher central blood pressure than men at same age. Postmenopausal hypertensive women had the highest values of indexes related to increased wave reflection. These findings highlight the influence of gender and the possible role of menopause on arterial stiffness.

## Abbreviations

AIx: Augmentation index
AIx75: Alx adjusted for a heart rate of 75
BP: Blood pressure
CV: Cardiovascular
DBP: Diastolic blood pressure
SBP: Systolic blood pressure
